# A Ranking Approach to Genomic Selection

**DOI:** 10.1371/journal.pone.0128570

**Published:** 2015-06-12

**Authors:** Mathieu Blondel, Akio Onogi, Hiroyoshi Iwata, Naonori Ueda

**Affiliations:** 1 NTT Communication Science Laboratories, Kyoto, Japan; 2 The University of Tokyo, Tokyo, Japan; Leibniz-Institute for Farm Animal Biology (FBN), GERMANY

## Abstract

**Background:**

Genomic selection (GS) is a recent selective breeding method which uses predictive models based on whole-genome molecular markers. Until now, existing studies formulated GS as the problem of modeling an individual’s breeding value for a particular trait of interest, i.e., as a regression problem. To assess predictive accuracy of the model, the Pearson correlation between observed and predicted trait values was used.

**Contributions:**

In this paper, we propose to formulate GS as the problem of ranking individuals according to their breeding value. Our proposed framework allows us to employ machine learning methods for ranking which had previously not been considered in the GS literature. To assess ranking accuracy of a model, we introduce a new measure originating from the information retrieval literature called normalized discounted cumulative gain (NDCG). NDCG rewards more strongly models which assign a high rank to individuals with high breeding value. Therefore, NDCG reflects a prerequisite objective in selective breeding: accurate selection of individuals with high breeding value.

**Results:**

We conducted a comparison of 10 existing regression methods and 3 new ranking methods on 6 datasets, consisting of 4 plant species and 25 traits. Our experimental results suggest that tree-based ensemble methods including McRank, Random Forests and Gradient Boosting Regression Trees achieve excellent ranking accuracy. RKHS regression and RankSVM also achieve good accuracy when used with an RBF kernel. Traditional regression methods such as Bayesian lasso, wBSR and BayesC were found less suitable for ranking. Pearson correlation was found to correlate poorly with NDCG. Our study suggests two important messages. First, ranking methods are a promising research direction in GS. Second, NDCG can be a useful evaluation measure for GS.

## Introduction

Traditional selective breeding, based on phenotypic or pedigree information, has led to much genetic improvement. Genomic selection (GS) [1] is a recent selective breeding method which uses predictive models based on whole-genome molecular markers. Compared to traditional marker-assisted selection methods, the key benefit of GS is that it uses markers covering the whole genome, thus making it possible to predict polygenic traits. With the constantly decreasing cost of marker technology, genotyping is currently less costly than phenotyping in applied plant breeding programs [2]. GS has already been adopted by dairy industries worldwide and is expected to double genetic gains for milk production and other traits [3]. GS can also accelerate selection cycles, since markers can be genotyped at birth or even before [4]. The effectiveness of GS has been confirmed in numerous studies, both for plant and animal breeding [2, 4–10].

Until now, GS has traditionally been formulated as the problem of predicting an individual’s breeding value for a given trait of interest; for instance, grain yield or or milk production. Therefore, GS was fundamentally formulated as a regression problem. To estimate a regression model, many different parametric and non-parametric methods were proposed in the literature including BayesA, BayesB, best linear unbiased prediction (BLUP) in the original work of [1], Bayesian lasso [7, 11, 12], a fast EM algorithm for the BayesB model called wBSR [13] and reproducing kernel Hilbert space (RKHS) regression [10, 14]. Recently, [15] compared popular methods from the GS literature with other machine learning methods including support vector regression, random forests and neural networks. Their results suggested that GS could be based on a reduced set of models such as Bayesian lasso, wBSR and random forests.

In this paper, we propose to formulate GS as a ranking problem. This is motivated by the fact that in order to select the most favorable individuals, we do not necessarily need to accurately predict breeding values. Instead, it is often sufficient to correctly rank individuals from most favorable to least favorable. As an example, consider the problem of selecting wheat lines according to their grain yield. In existing studies, this would be formulated as the problem of predicting grain yield from genotypes. In our approach, this is formulated instead as the problem of correctly ranking wheat lines in order of decreasing grain yield. Our proposed framework allows us to employ machine learning methods for ranking which had previously never been considered in the GS literature. Until now, the predictive accuracy of a model was typically assessed using the Pearson correlation between observed trait values and the predicted trait values (a.k.a. genomic estimated breeding values, GEBV). However, our experiments show that Pearson correlation may correlate poorly with ranking accuracy. In this paper, we introduce a new measure originating from the information retrieval literature called normalized discounted cumulative gain (NDCG) [16]. NDCG rewards more strongly models which assign high rank to individuals with high breeding value. In addition, NDCG focuses on the top individuals in the ranking, while Pearson correlation treats all individuals uniformly. Therefore, NDCG reflects a prerequisite objective in selective breeding: accurate selection of the top individuals with highest breeding value.

## Regression-based genomic selection

### General approach

In this section, we first review the two-phase approach usually taken by traditional regression-based GS methods.

In the **model estimation** phase (also known as **training phase**), a reference population, which has been genotyped and whose trait values are known, is used to estimate a statistical model of the relationship between genotypes and the trait. For a reference population of size *n*, we denote the genotypes by ***x***_1_, …, ***x***_*n*_, where ***x***_*i*_ ∈ 𝓧 ⊆ ℝ^*p*^, and the associated trait values by *y*_1_, …, *y*_*n*_, where *y*_*i*_ ∈ 𝓨 ⊆ ℝ. The number *p* indicates the number of molecular markers (e.g., DArT, SNP, …) used for genotyping. For simplicity, in the remainder of this paper, we use ***x***_*i*_ to refer to both individual *i* and its vector representation after genotyping. Likewise, phenotypic values or estimated breeding values will simply be referred to as “trait values”. Trait values of the reference population are typically obtained by field testings, which are expensive and time-consuming. Therefore, *n* is usually small. On the other hand, the number of markers *p* is usually large for genome-wide genotyping. Therefore, *n* is usually much smaller than *p*. This is known as the *n* ≪ *p* problem. Existing GS approaches estimate a regression model *h*: ℝ^*p*^ → ℝ such that **h**(**x**_*i*_) ≈ *y*_*i*_ for all *i* ∈ {1, …, *n*}. We briefly review common approaches for estimating *h* further below. In the following, we denote by *X* the *n* × *p* matrix which gathers individuals ***x***_1_, …, ***x***_*n*_, by **y** the *n*-dimensional vector which gathers their true trait values *y*_1_, …, *y*_*n*_ and by $\hat{\text{y}}$ the *n*-dimensional vector which gathers the predicted values ${\hat{y}}_{1},\ldots ,{\hat{y}}_{n}$, where ${\hat{y}}_{i}=h({\text{x}}_{i})$.

In the **candidate selection** phase, predicted trait values are computed using the fitted model for candidate individuals to be selected. We denote the genotypes of *m* candidates by ${\bar{\text{x}}}_{1},\ldots ,{\bar{\text{x}}}_{m}$. Contrary to the reference population, the true trait values of the candidates are not known. Throughout this paper, we assume that the individuals from the reference population ***x***_1_, …, ***x***_*n*_ and candidate individuals ${\bar{\text{x}}}_{1},\ldots ,{\bar{\text{x}}}_{m}$ are sampled i.i.d. (independent and identically distributed) from the same (unknown) distribution.

### Traditional evaluation measures

Model evaluation is the task of evaluating how good a model is and is crucial to choose the best model among several possible choices. In the GS literature, model evaluation has traditionally been carried out using mainly two measures: mean squared error (MSE) and Pearson correlation. MSE is defined by

$$\begin{array}{c} \text{MSE}\left(y,\hat{y}\right)=\frac{1}{n}\sum_{i=1}^{n}{\left(y_{i}-{\hat{y}}_{i}\right)}^{2}. \end{array}$$

The model is better when $\text{MSE}(\text{y},\hat{\text{y}})$ is lower and perfect fit is achieved when $\text{MSE}(\text{y},\hat{\text{y}})=0$. Obviously, $\text{MSE}(\text{y},\hat{\text{y}})=0$ if ${\hat{y}}_{i}=y_{i}$ for all *i*. In other words, a model achieves zero error if it predicts perfectly all trait values. Note that this can only happen when heritability is one and all genetic variance is explained by the markers.

A more commonly used measure in the GS literature is the Pearson correlation between observed and predicted trait values. It is defined by

$$\begin{array}{c} r\left(y,\hat{y}\right)=\frac{\sum_{i=1}^{n}\left(y_{i}-\mu_{y}\right)\left({\hat{y}}_{i}-\mu_{\hat{y}}\right)}{\sqrt{\sum_{i=1}^{n}{\left(y_{i}-\mu_{y}\right)}^{2}\sum_{i=1}^{n}{\left({\hat{y}}_{i}-\mu_{\hat{y}}\right)}^{2}}}, \end{array}$$

where $\mu_{y}=\frac{1}{n}\sum_{i=1}^{n}y_{i}$ and $\mu_{\hat{y}}=\frac{1}{n}\sum_{i=1}^{n}{\hat{y}}_{i}$. The model is better when $r(\text{y},\hat{\text{y}})$ is higher and perfect correlation is achieved when $r(\text{y},\hat{\text{y}})=1$. Contrary to MSE, correlation does not require to accurately predict trait values. Indeed, it can be seen that $r(\text{y},\hat{\text{y}})=1$ if there exists *a* > 0 and *b* such that ${\hat{y}}_{i}=ay_{i}+b$ for all *i*. In other words, the set of points ${\left\{\left(y_{i},{\hat{y}}_{i}\right)\right\}}_{i=1}^{n}$ must be collinear in order to achieve perfect correlation. Again, perfect correlation can only happen when heritability is one and all genetic variance is explained by the markers. When heritability is less than one, correlation has an upper limit which is equal to the square root of heritability. If the proportion of genetic variance that markers can explain is less than one, the upper limit decreases from the square root of heritability.

### Overview of popular regression models

In this section, we briefly describe various regression models, focusing on the most popular ones in the GS literature.

#### Ridge and RKHS regression

One of the first methods proposed for genomic selection was ridge regression, which is equivalent to best linear unbiased prediction (BLUP) in the context of mixed models [1]. Let *I*_*p*_ be the identity matrix of size *p* × *p*. The basic model is ***y*** = *X* ***β***+*ϵ*, where $\text{β}∼Normal(0,\sigma_{u}^{2}I_{p})$ and $\varepsilon ∼Normal(0,\sigma_{e}^{2})$. The solution for the marker effects can be obtained by ***β*** = (*X* ^T^ *X*+*λI*_*p*_)^−1^ *X* ^T^ ***y***, where $\lambda =\sigma_{e}^{2}/\sigma_{u}^{2}$ is the ratio between the residual and marker variances. Predictions can be computed by *h*(***x***) = ***β*** ^T^ ***x***. The representer theorem [17, 18] guarantees that ***β*** can be written as a linear combination of the data, i.e., ***β*** = *X* ^T^ **α** for some **α** ∈ ℝ^*n*^. This has two important implications. The first is that we can equivalently compute ***β*** by ***β*** = *X* ^T^ **α** = *X* ^T^(*XX* ^T^+*λI*_*n*_)^−1^ ***y***. The main difference is that we now need to invert a *n* × *n* matrix instead of a *p* × *p* one. This is advantageous in GS because we usually have *n* ≪ *p*. The second implication is that ridge regression can now be “kernelized” by using the identity $\hat{\text{y}}=X\text{β}=XX^{\text{T}}\text{α}=K\text{α}$, where *K* = *XX* ^*T*^ and **α** = (*K*+*λI*_*n*_)^−1^ ***y***. In practice, *K* can be replaced by any positive semidefinite kernel matrix with elements *K*_*ij*_ = κ(***x***_*i*_,***x***_*j*_), where κ is the corresponding kernel function. Predictions can then be computed by $h(\text{x})=\sum_{i=1}^{n}\alpha_{i}\kappa (\text{x},{\text{x}}_{i})$. The result is known as kernel ridge regression in the machine learning literature and as RKHS regression in the GS literature. The elements *K*_*ij*_ correspond to inner products in a high-dimensional (possibly infinite) space called reproducing kernel Hilbert space (RKHS). This allows to model non-linear relationships between *X* and ***y***. RKHS regression is equivalent to ridge regression when using a linear kernel.

#### Bayesian lasso (BL)

Bayesian lasso [11] is the Bayesian counterpart of the lasso [19]. Following the parameterization of [20], the effect of marker *j*, *β*_*j*_, was assumed to follow a hierarchical prior distribution,

$$\begin{array}{c} Normal(\beta_{j}|0,1/\tau_{j}^{2}\tau_{0}^{2})InvGamma(\tau_{j}^{2}|1,\lambda_{B}^{2}/2), \end{array}$$

where *Normal* and *InvGamma* indicate the normal and inverse gamma distributions, respectively, $\tau_{j}^{2}$ determines the shrinkage magnitude for *β*_*j*_, $1/\tau_{0}^{2}$ is the residual variance and $\lambda_{B}^{2}$ is a hyper-parameter that defines the distribution of $\tau_{j}^{2}$. We modified the method of [20] such that marker effects were conditional on the residual variance (precision), as in [11]. The prior distribution of $\lambda_{B}^{2}$ is the gamma distribution *Gamma*(*ϕ*, *ω*), where *ϕ* and *ω* are the shape and rate parameters, respectively.

#### Extended Bayesian lasso (EBL)

In the EBL [21], $\lambda_{B}^{2}$ is replaced with $\delta^{2}\eta_{j}^{2}$, where *δ* ^2^ is a global shrinkage factor and $\eta_{j}^{2}$ is a shrinkage factor for marker *j*. The priors used are *Gamma*(*ϕ*, *ω*) for *δ* ^2^ and *Gamma*(*ψ*, *θ*) for $\eta_{j}^{2}$.

#### Weighted Bayesian shrinkage regression (wBSR)

wBSR [13] uses the indicator variable *γ*_*j*_ to determine whether the marker effect *β*_*j*_ is included in the regression model (*γ*_*j*_ = 1) or not (*γ*_*j*_ = 0). A prior Bernoulli distribution, *Bernoulli*(*γ*_*j*_∣*π*), is assumed for *γ*_*j*_. The marker effect *β*_*j*_ is assumed to follow a hierarchical prior distribution,

$$\begin{array}{c} Normal(\beta_{j}|0,\sigma_{j}^{2})InvChi2(\sigma_{j}^{2}|\nu ,S^{2}), \end{array}$$

where *InvChi2* indicates a scaled inverse chi-squared distribution, *ν* is the degree of freedom and *S* ^2^ is the scaling parameter.

#### BayesC

In BayesC [22], *β*_*j*_ is assumed to follow a spike and slab prior distribution,

$$\begin{array}{c} p\left(\beta_{j}\right)∼\{\begin{array}{cc} Normal(\beta_{j}|0,\sigma^{2}) & \text{if}\;\rho_{j}=1\\ 0 & \text{if}\;\rho_{j}=0 \end{array}, \end{array}$$

where *ρ*_*j*_ is an indicator variable. The prior distributions used for *ρ*_*j*_ and *σ* ^2^ are *Bernoulli*(*ρ*_*j*_∣*π*) and *InvChi*2(*σ* ^2^∣*ν*, *S* ^2^), respectively.

#### Stochastic search variable selection (SSVS)

SSVS [23] assumes the following prior distribution for *β*_*j*_,

$$\begin{array}{c} p\left(\beta_{j}\right)∼\{\begin{array}{cc} Normal(\beta_{j}|0,\sigma^{2}) & \text{if}\;\rho_{j}=1\\ Normal(\beta_{j}|0,c\sigma^{2}) & \text{if}\;\rho_{j}=0 \end{array}, \end{array}$$

where *c* < 1 determines the relative magnitude of the variances of the two normal distributions.

#### Bayesian mixture regression model (MIX)

For the prior distribution of *β*_*j*_, MIX [24] assumes a mixture of two normal distributions with variances independent of one another:

$$\begin{array}{c} p\left(\beta_{j}\right)∼\{\begin{array}{cc} Normal(\beta_{j}|0,\sigma_{1}^{2}) & \text{if}\;\rho_{j}=1\\ Normal(\beta_{j}|0,\sigma_{0}^{2}) & \text{if}\;\rho_{j}=0 \end{array}. \end{array}$$

In [24], the prior distributions of $\sigma_{1}^{2}$ and $\sigma_{0}^{2}$ were $InvChi2(\sigma_{1}^{2}|\nu ,S^{2})$ and $InvChi2(\sigma_{0}^{2}|\nu ,S^{2})$. We modified the prior of $\sigma_{0}^{2}$ to $InvChi2(\sigma_{0}^{2}|\nu ,cS^{2})$ so as to encourage clustering of markers according to the magnitude of their effects.

#### Random forests (RF)

RF [25] are an ensemble algorithm based on randomized regression trees. In RF, each tree is built from a sample drawn with replacement (i.e., a bootstrap sample) from the training set. The final prediction is computed by averaging the predictions of all trees in the forest. This procedure is known to improve the bias-variance trade-off of regression trees and leads to highly accurate predictions. To further reduce overfitting, two heuristics are typically applied in RF. The first heuristic consists, when splitting a node during the construction of a tree, in selecting the best split from a random subset of the features (“max_features”). This both improves accuracy and reduces training time. The second heuristic consists in limiting the maximum depth of the regression trees (“max_depth”). This ensures that trees are not too complicated. Although careful tuning of these two parameters can improve accuracy, we find that RF are pretty robust to their choice.

#### Gradient boosted regression trees (GBRT)

In gradient boosting [26], an ensemble of regression models is built in a stage-wise fashion so as to minimize a differentiable loss function. GBRT refers to gradient boosting when the models are regression trees. GBRT starts with a base model *h*_0_. For regression with squared loss, a common choice is the base model which always outputs the training set’s target mean irrespective of ***x***: $h_{0}(\text{x})=\frac{1}{n}\sum_{i=1}^{n}y_{i}$. Subsequently, GBRT incrementally adds new trees *h*_1_, …, *h*_*M*_ to obtain an ensemble $h(\text{x})=\sum_{s=0}^{M}\alpha_{s}h_{s}(\text{x})$. For the squared loss, the tree *h*_*s*_ at stage *s* is fitted against the residuals of the ensemble so far *e*_1_, …, *e*_*n*_, where $e_{i}=y_{i}-\sum_{r=1}^{s-1}\alpha_{r}h_{r}(\text{x})$. For other differentiable loss functions, residuals are replaced with the negative gradient, which [26] calls “pseudo-responses”. At each stage *s*, GBRT finds *α*_*s*_ by line search and multiply the result by a small learning rate, typically between 10^−3^ and 1, to avoid overfitting. To further reduce overfitting, the same heuristics as RF can be applied (“max_features” and “max_depth”). Although past GS works did not consider GBRT, we include it in our comparison since it achieved among the leading results in the Yahoo! learning to rank challenge [27].

## Ranking-based genomic selection

### General approach

Similarly to existing regression-based approaches, our approach is broken down into a model estimation phase and a candidate selection phase. The main difference is that, in our approach, we do not impose that the model *h* satisfy *h*(***x***_*i*_) ≈ *y*_*i*_. Instead, in our approach, *h* is a **scoring** function: it assigns a score to each candidate. The scores are then used to determine a **ranking** of the candidates. In this ranking framework, GS can be summarized by the following two phases:

1. **Model estimation.** Using the reference population, **estimate** a scoring function *h* which can be used for ranking. Intuitively, a perfect scoring function would satisfy *h*(***x***_(1)_) ≥ *h*(***x***_(2)_) ≥ … ≥ *h*(***x***_(*n*)_) if *y*_(1)_ ≥ *y*_(2)_ ≥ … ≥ *y*_(*n*)_.
2. **Candidate selection.** Using *h*, **rank** *m* candidates ${\bar{\text{x}}}_{1},{\bar{\text{x}}}_{2},\ldots ,{\bar{\text{x}}}_{m}$ by decreasing scores. We denote the ranked candidates by ${\bar{\text{x}}}_{\left(1\right)}{≽}_{h}{\bar{\text{x}}}_{\left(2\right)}{≽}_{h}\ldots {≽}_{h}{\bar{\text{x}}}_{\left(m\right)}$, where ***x***_*i*_ ≽_*h*_ ***x***_*j*_ means *h*(***x***_*i*_) ≥ *h*(***x***_*j*_). Finally, **select** the top *k* candidates ${\bar{\text{x}}}_{\left(1\right)}{≽}_{h}{\bar{\text{x}}}_{\left(2\right)}{≽}_{h}\ldots {≽}_{h}{\bar{\text{x}}}_{\left(k\right)}$ for further field testing. Typically, the number of selected candidates *k* is chosen much smaller than the total number of candidates *m*, i.e., *k* ≪ *m*.

Our ranking-based formulation naturally captures a prerequisite objective in selective breeding: accurate selection of individuals with high breeding value. In this section, since we do not impose that *h*(***x***_*i*_) ≈ *y*_*i*_, $\hat{\text{y}}$ does not necessarily represent predicted trait values. Instead, $\hat{\text{y}}$ is the *n*-dimensional vector which gathers predicted scores ${\hat{y}}_{1},\ldots ,{\hat{y}}_{n}$, where ${\hat{y}}_{i}=h({\text{x}}_{i})$, from which individuals can be sorted in decreasing order.

### Evaluation measures for global ranking

As we explained previously, the Pearson correlation does not require to accurately predict trait values. It only requires the set of points ${\left\{\left(y_{i},{\hat{y}}_{i}\right)\right\}}_{i=1}^{n}$ to be collinear. In that sense, the Pearson correlation can be seen as a ranking measure. However, the collinearity requirement may sometimes be too strict. To illustrate the problem, consider the case when ***y*** = [3.5, 2.8, 1.2] and $\hat{\text{y}}=[10.3,3.7,0.1]$. In this example, the model achieves perfect ranking since *y*_1_ ≥ *y*_2_ ≥ *y*_3_ and ${\hat{y}}_{1}\geq {\hat{y}}_{2}\geq {\hat{y}}_{3}$. However, because the true and predicted trait values are not perfectly collinear, the correlation is only equal to $r(\text{y},\hat{\text{y}})=0.92$. This example shows that it is not necessary to achieve perfect correlation to achieve perfect ranking. In this section, we present two related measures for global ranking evaluation that do not assume collinearity: pairwise accuracy and Kendall’s *τ*.

Given the reference trait values ***y***, we define the preference set as *P*(***y***) = {(*i*, *j*):**y**_*i*_ > *y*_*j*_}. Intuitively, if (*i*, *j*) ∈ *P*(***y***), then ***x***_*i*_ is preferred to ***x***_*j*_ (e.g., ***x***_*i*_ has higher grain yield than ***x***_*j*_). Given the predicted scores $\hat{\text{y}}$, we define the set of concordant pairs as $C(\text{y},\hat{\text{y}})=\{(i,j)\in P(\text{y}):{\hat{y}}_{i}>{\hat{y}}_{j}\}$ and the set of discordant pairs as $D(\text{y},\hat{\text{y}})=\{(i,j)\in P(\text{y}):{\hat{y}}_{i}<{\hat{y}}_{j}\}$. Pairs in the set $T(\text{y},\hat{\text{y}})=\{(i,j)\in P(\text{y}):{\hat{y}}_{i}={\hat{y}}_{j}\}$ are neither concordant nor discordant.

Pairwise accuracy (c.f., e.g., [28]) is simply defined as the proportion of concordant pairs:

$$\begin{array}{c} \text{pairwise\_accuracy}\left(y,\hat{y}\right)=\frac{\left|C(y,\hat{y})\right|}{\left|P(y)\right|}, \end{array}$$

where ∣*S*∣ is the cardinality of the set *S*. Pairwise accuracy is 0 when not a single pair was concordant and is 1 when all pairs were concordant. For binary trait values, pairwise accuracy is exactly equivalent to the area under the ROC curve (AUC) and is closely related to the Mann-Whitney-Wilcoxon statistic [29]. Pairwise ranking algorithms such as RankSVM [30], RankBoost [31] and RankNet [32] maximize an upper-bound on pairwise accuracy.

Another commonly used measure is Kendall’s *τ*[33]:

$$\begin{array}{c} \tau \left(y,\hat{y}\right)=\frac{|C\left(y,\hat{y}\right)|-|D\left(y,\hat{y}\right)|}{\left|P(y)\right|} \end{array}$$

Intuitively, Kendall’s *τ* is the difference between the ratio of concordant pairs and the ratio of discordant pairs. Kendall’s *τ* is always between −1 and 1.

Assuming $T(\text{y},\hat{\text{y}})$ is an empty set (which is likely to be the case, since $\hat{\text{y}}$ is a continuous vector), Kendall’s *τ* and pairwise accuracy are directly related by the following formula:

$$\begin{array}{c} \tau \left(y,\hat{y}\right)=\frac{2|C(y,\hat{y})|}{\left|P(y)\right|}-1=2\times \text{pairwise\_accuracy}\left(y,\hat{y}\right)-1. \end{array}$$

Therefore, algorithms which are designed to maximize pairwise accuracy will also maximize Kendall’s *τ*.

### Evaluation measures for top-*k* ranking

One issue with pairwise accuracy and Kendall’s *τ* is that they treat all pairs equally. Intuitively, a good ranking evaluation measure should fulfill two requirements. First, it should reward more strongly assigning a high rank to individuals with high breeding value. Second, it should focus on the top *k* individuals in the ranking. Oftentimes, it does not matter if a model cannot correctly order individuals with low breeding value. Instead, it is sufficient to rank as many individuals with high breeding value as possible at the top. In this section, we introduce two measures that fulfill the above two requirements: discounted cumulative gain (DCG) [16] and its normalized version (NDCG). In the information retrieval (IR) literature, NDCG has been popularly used to measure the ability of search engines to retrieve highly relevant documents in the top search results. In this paper, we use NDCG to measure the ability of GS models to select the top *k* individuals with highest breeding value.

To introduce discounted cumulative gain (DCG), we first note that any predicted score vector $\hat{\text{y}}=[{\hat{y}}_{1},\ldots ,{\hat{y}}_{n}]$ induces a permutation ***π*** = [*π*_1_, …, *π*_*n*_] of [1, …, *n*] such that the scores are sorted in decreasing order: ${\hat{y}}_{\pi_{1}}\geq {\hat{y}}_{\pi_{2}}\geq \ldots \geq {\hat{y}}_{\pi_{n}}$. Given the reference trait values **y** = [*y*_1_, …, *y*_*n*_] and any such permutation ***π***, the DCG at position *k* (we assume *k* ≤ *n*) is defined by

$$\begin{array}{c} \text{DCG@}k\left(y,\pi \right)=\sum_{i=1}^{k}g\left(y_{\pi_{i}}\right)d\left(i\right). \end{array}$$

Here, *g*(*y*) is a monotonically increasing gain function and *d*(*i*) is a monotonically decreasing discount function. Common choices for the gain function are *g*(*y*) = *y* (linear gains) and *g*(*y*) = 2^*y*^−1 (exponential gains). For the discount function, a common choice is $d(i)=\frac{1}{{\text{log}}_{2}(i+1)}$. Intuitively, a model obtains the highest possible DCG@*k* when the order of the predicted scores $\hat{\text{y}}$ agrees with the order of the true observed traits ***y***.

DCG can be difficult to interpret because its values are unbounded. In practice, the normalized DCG (NDCG) is often used instead. If we define by *π*(***y***) = [*π*(***y***)_1_, …, *π*(***y***)_*n*_] a permutation of [1, …, *n*] for sorting ***y*** in decreasing order, i.e., *y*_*π*(***y***)_1__ ≥ … ≥ *y*_*π*(***y***)_*n*__, then NDCG at position *k* is defined by

$$\begin{array}{c} \text{NDCG@}k\left(y,\hat{y}\right)=\frac{\text{DCG@}k(y,\pi \left(\hat{y}\right))}{\text{DCG@}k(y,\pi (y))}. \end{array}$$

Intuitively, NDCG is simply the ratio between the DCG score of the predicted ranking and the DCG score of the ideal ranking. NDCG is easier to interpret than DCG because its values are always between 0 and 1, assuming $\text{y}\in {\mathbb{R}}_{+}^{n}$.

In our experiments, we also report results of Mean NDCG@*K*, which is simply the mean of NDCG scores from *k* = 1 to *k* = *K*:

$$\begin{array}{c} \text{Mean}\;\text{NDCG@}K\left(y,\hat{y}\right)=\frac{1}{K}\sum_{k=1}^{K}\text{NDCG@}k\left(y,\hat{y}\right). \end{array}$$

We discuss the choices of the position *k*, gain function *g*(*y*) and discount function *d*(*i*) in the “Discussion” section.

### Background on “learning to rank”

Estimating a ranking model, commonly known as “learning to rank”, has attracted a great deal of research in the machine learning community. Intuitively, when formulating GS as a ranking problem, we should estimate a model so as to directly maximize a ranking accuracy measure of interest, such as NDCG. Unfortunately, this turns out to be a non-convex problem that can be NP-hard [34]. To solve this problem, learning to rank approaches replace the true loss function by an easier to optimize one called surrogate loss function. Learning to rank approaches are typically divided into three categories depending on the type of surrogate loss function used.

**Pointwise** approaches involve a surrogate loss function on individual samples ***x***_*i*_. They typically reduce ranking to either regression, classification or ordinal regression/classification. It was shown that DCG errors are bounded by regression [34] and classification [35] errors.

**Pairwise** approaches involve a surrogate loss function on pairs of samples (***x***_*i*_,**x**_*j*_). Their main idea is that if *y*_*i*_ > *y*_*j*_, then the model *h* does not need to predict *y*_*i*_ and *y*_*j*_ accurately: it only needs to respect the relative order *h*(***x***_*i*_) > *h*(**x**_*j*_). RankSVM [30], RankBoost [31] and RankNet [32] are based on pairwise versions of the hinge, exponential and logistic surrogate loss functions, respectively.

**Listwise** approaches involve a surrogate loss function or algorithm based on a list of samples. Some listwise methods such as LambdaMART [36] can optimize top-*k* ranking accuracy directly.

For more details on “learning to rank”, we refer the reader to [37, 38].

### Overview of ranking models

Our ranking-based formulation allows us to employ machine learning methods for ranking which had never been considered in the GS literature before. For our experiments, we chose three representative methods of the pointwise, pairwise and listwise categories.

#### McRank (pointwise)

McRank [35] is a method for indirectly optimizing NDCG through multiple classification. This is motivated by the fact that classification can be an easier task than regression. Suppose that the traits can only take on a finite number *B* of values, i.e., 𝓨 = {*b*_1_, *b*_2_, …, *b*_*B*_}, where *b*_*r*_ ∈ ℝ. If that is not the case, we can always discretize the trait values (of the training set only), as explained in our experiments. The main idea of McRank is to rank candidates according to their expected trait value, which can be computed by $h(\text{x})=\sum_{r=1}^{B}Pr(y=b_{r}|\text{x})b_{r}$. McRank exists in two variants: multiclass and ordinal McRank. The variants differ in how they compute the *Pr*(*y* = *b*_*r*_∣***x***) probabilities.

**Multiclass McRank** models the *Pr*(*y* = *b*_*r*_∣***x***) class probabilities using a probabilistic classifier. Any probabilistic classifier (e.g., logistic regression) can in theory be used. Although the original McRank paper used gradient boosting as classifier, in this paper, we used random forests, since they worked better in our experiments. Unfortunately, multiclass McRank completely ignores the natural ordering *b*_1_ ≤ *b*_2_ ≤ … ≤ *b*_*B*_.

**Ordinal McRank** addresses this problem as follows. Notice that *Pr*(*y* = *b*_*r*_∣***x***) = *Pr*(*y* ≤ *b*_*r*_∣***x***)−*Pr*(*y* ≤ *b*_*r*−1_∣***x***). In other words, we can model class probabilities *Pr*(*y* = *b*_*r*_∣***x***) using cumulative probabilities *Pr*(*y* ≤ *b*_*r*_∣***x***) and *Pr*(*y* ≤ *b*_*r*−1_∣***x***). The advantage is that this takes into account the natural ordering *b*_1_ ≤ *b*_2_ ≤ … ≤ *b*_*B*_. Cumulative probabilities *Pr*(*y* ≤ *b*_*r*_∣***x***) can easily be modeled as follows. First, we partition the training data into two groups {*y*_*i*_ ≤ *b*_*r*_} (positive class) and {*y*_*i*_ ≥ *b*_*r*+1_} (negative class). Using this partition, we can then train a two-class probabilistic classifier. The probability of the positive class gives *Pr*(*y* ≤ *b*_*r*_∣***x***). Again, although any probabilistic classifier can be used, we used random forests since they performed best in our experiments.

#### RankSVM (pairwise)

Recall that we previously defined the preference set as *P*(***y***) = {(*i*, *j*):*y*_*i*_ > *y*_*j*_}. The main idea of RankSVM [30] is to use the support vector machine (SVM) framework in order to find a model *h*(***x***) such that *h*(***x***_*i*_) > *h*(***x***_*j*_) for all (*i*, *j*) ∈ *P*(***y***). In this paper, we consider the kernelized version of RankSVM [39], which minimizes the objective

$$\begin{array}{c} \underset{\alpha \in {\mathbb{R}}^{n}}{\text{minimize}}\;f\left(\alpha \right)\equiv \frac{\lambda }{2}\alpha^{T}K\alpha +\sum_{\left(i,j)\in P(y\right)}\max{\left(0,1-\alpha^{T}K_{i}+\alpha^{T}K_{j}\right)}^{r}, \end{array}$$

where *λ* is a regularization parameter, *K* ∈ ℝ^*n* × *n*^ is a kernel matrix with elements *K*_*ij*_ = *κ*(***x***_*i*_,***x***_*j*_), *K*_*i*_ ∈ ℝ^*n*^ is the *i* ^th^ column of *K* and *r* ∈ {1, 2}. Once **α** has been obtained, the model $h(\text{x})=\sum_{i=1}^{n}\alpha_{i}\kappa (\text{x},{\text{x}}_{i})$ can be used to sort candidates in decreasing order. Using the kernelized version of RankSVM has the same advantages as RKHS regression. First, the model is non-linear if we use a non-linear kernel. This allows to model non-linear relationships. Second, the optimization problem is *n*-dimensional instead of *p*-dimensional, which is advantageous in GS. When *r* = 2, the RankSVM objective is differentiable and can thus be solved by gradient methods such as the conjugate gradient method or limited-memory BFGS (L-BFGS) [40]. The gradient expression necessary to run these methods is given by

$$\begin{array}{c} \nabla f\left(\alpha \right)=\lambda K\alpha +2\sum_{(i,j)\in P}\max(0,1-\alpha^{T}K_{i}+\alpha^{T}K_{j})\left(K_{j}-K_{i}\right). \end{array}$$

The global solution, which is unique, is guaranteed to be found, since RankSVM has a strictly convex objective [39]. When *r* = 1, the conjugate gradient and L-BFGS methods cannot be used, since the RankSVM objective is not differentiable everywhere. Instead, it is possible to solve the objective using the subgradient method.

#### LambdaMART (listwise)

LambdaMART [36, 41] is a method which builds upon GBRT (c.f. overview of regression models) to optimize NDCG@k. It achieved the leading results in the Yahoo! learning to rank challenge [27]. As explained previously, GBRT incrementally builds an ensemble of *M* regression trees $h(\text{x})=\sum_{s=0}^{M}\alpha_{s}h_{s}(\text{x})$ by adding on each stage a regression tree *h*_*s*_ fitted against “pseudo-responses”, the negative gradient of the objective function. To deal with the discontinuity of the NDCG objective, LambdaMART uses an approximation of the negative gradient called *λ*-gradient. GBRT is also known as MART (multiple additive regression trees), hence the name LambdaMART. Let us define the following expressions on stage *s*:

$$\begin{array}{ccc} \lambda_{ij} & = & \text{sign}\left(o_{ij}\right)|\Delta {\text{NDCG}}_{ij}\frac{\partial C_{ij}}{\partial o_{ij}}|\;\lambda \text{-gradient}\;\text{for}\;\text{the}\;\left(x_{i},x_{j}\right)\;\text{pair}\\ \frac{\partial C_{ij}}{\partial o_{ij}} & = & \frac{1}{1+\exp(o_{ij})}\;\text{cross-entropy}\;\text{derivative}\\ o_{ij} & = & {\hat{y}}_{i}-{\hat{y}}_{j}\;\text{prediction}\;\text{score}\;\text{difference}\\ \Delta {\text{NDCG}}_{ij} & = & \text{NDCG}\;\text{gained}\;\text{by}\;\text{swapping}\;x_{i}\;\text{and}\;x_{j}\\ {\hat{y}}_{i} & = & \sum_{r=0}^{s-1}\alpha_{r}h_{r}\left(x_{i}\right)\;\text{prediction}\;\text{score}\;\text{up}\;\text{to}\;\text{stage}\;s-1. \end{array}$$

Then, the *λ*-gradient on stage *s* is an *n*-dimensional vector whose elements are defined by $\lambda_{i}=\sum_{j=1}^{n}\lambda_{ij}$. The *λ*_*ij*_ can be interpreted as follows. If ***x***_*i*_ has higher breeding value than ***x***_*j*_, then ***x***_*j*_ will get a push downwards of strength ∣*λ*_*ij*_∣. Otherwise, ***x***_*j*_ will get a push upwards of strength ∣*λ*_*ij*_∣. However, if **x**_*i*_ and **x**_*j*_ are both not ranked in the top-*k* elements, then, by the definition of NDCG, there will be no gain from swapping them. Therefore, ΔNDCG_*ij*_ = *λ*_*ij*_ = 0 in this case. For a detailed derivation and discussion of the *λ*-gradient, see [41]. To reduce overfitting, the same techniques as RF and GBRT can be used.

#### Complexity comparison

We now briefly compare the computational complexity of training algorithms for ranking and regression. We assume that the number of samples *n* is much smaller than the number of markers *p*, as is usually the case in GS. For RKHS regression (a.k.a. kernel ridge regression), we pre-compute the kernel matrix *K*, which takes *O*(*n* ^2^ *p*). Once this is done, a closed form solution can be obtained by solving a system of linear equations, which takes *O*(*n* ^3^). Alternatively, an approximate solution can be obtained by any gradient solver, such as the conjugate gradient method or limited-memory BFGS (L-BFGS). In this case, the main cost per iteration comes from computing the gradient, which takes *O*(*n* ^2^). For kernel RankSVM, we also pre-compute the kernel matrix. Since a closed form solution is not available, we use a gradient method. The main cost per iteration stems from computing the gradient, which takes *O*(*n* ^2^), the same as for RKHS regression. For random forests, GBRT, McRank and LambdaMART, the computational cost is proportional to the number of trees in the ensemble. Tree induction takes *O*(*p*′*n* log^2^ *n*) in average and *O*(*p*′*n* ^2^log*n*) in worst case, where *p*′ ≤ *p* is the number of markers considered for node splitting [42]. For LambdaMART, each tree needs to be fitted against the *λ*-gradient. Computing the *λ*-gradient takes *O*(*kn*), where *k* is the parameter used for NDCG@k.

## Experimental results

### Datasets

We evaluated the validity of our ranking approach using the following 6 datasets.

#### Arabidopsis

This dataset comprises 422 lines of *Arabidopsis thaliana* developed by INRA [43]. We chose 3 traits, flowering time in short days (FLOSD), shoot dry matter in non-limiting nitrogen conditions (DM10) and shoot dry matter in limiting nitrogen conditions (DM3), which were also selected in [15, 44]. We excluded 5 lines which were not evaluated for these traits. Marker genotypes were available for 69 SSRs. We imputed the missing genotypes using the R package *qtl*[45]. The dataset is available at http://publiclines.versailles.inra.fr/page/33.

#### Barley

The Barley-CAP project evaluated the grain yield of 432 barley lines in Aberdeen, Idaho (trial name: NSGC_2012_NormN_Irr_Aberdeen). Among them, 381 lines were genotyped using 3945 SNPs. We imputed missing genotypes using BEAGLE [46]. The dataset is available at http://triticeaetoolbox.org.

#### Maize

This dataset, used in the study of [10], comprises 264 maize lines genotyped using 1076 SNP markers. We imputed missing values using BEAGLE [46]. The dataset is provided as example data in SelectionTools [47], available at http://www.uni-giessen.de/cms/fbz/fb09/institute/pflbz2/population-genetics/downloads.

#### Rice

This dataset consists of 395 lines genotyped with 1311 SNPs [48]. We imputed missing genotypes using BEAGLE [46]. Among these lines, phenotypic values of a total of 34 traits were available for 383 lines, with some missing records [49]. We chose 335 lines without missing records in 14 traits. The traits were flowering time, flag leaf length, flag leaf width, number of panicles per plant, plant height, panicle length, primary panicle branch number, number of seeds per panicle, number of florets per panicle, seed length, seed width, seed volume, seed surface area and amylose content. The dataset is available at http://www.ricediversity.org/data/index.cfm.

#### Wheat (CIMMYT)

This dataset comprises 599 wheat lines developed by the CIMMYT Global Wheat Breeding program [10]. Trait values correspond to grain yield evaluated in 4 different environments. Wheat lines were genotyped using 1447 DArT (Diversity Array Technology) markers. Markers may take on the values 1 or 0, indicating their presence or absence. Markers with allele frequency less than 0.05 or greater than 0.95 were removed. The total number of markers retained after this processing was 1279. The dataset is provided as part of the R package *BLR*.

#### Wheat (Pérez-Rodríguez)

This dataset, used in the study of [50], consists of 306 lines genotyped with 1695 DArT markers. Phenotypic values for two traits, grain yield and days to heading, were available for these lines. The dataset is available at the same URL as for Maize data.

For SSR and SNP markers, genotypes were encoded by 0 (AA), 1 (AB), and 2 (BB). For DArT markers, the presence or absence of an allele was encoded by 0 and 1 for Wheat (CIMMYT) and by 0 and 2 for Wheat (Pérez-Rodríguez).

For datasets comprised of several traits, we build one model per trait and report the averaged evaluation scores.

All traits described above are inherently non-negative quantities. In the case of the Wheat (CIMMYT) dataset, grain yield values were centered so as to have zero mean prior to public release of the dataset. As a result, the dataset contains negative yield values. In order to avoid negative NDCG scores, we converted the yield values back to positive values. To do so, since the original centering was not known to us, we simply shifted the yield values such that the smallest value in the entire dataset is zero.

### Experimental setup

#### Cross-validation setup

To estimate the generalization performance of different models, i.e., the ranking accuracy on new candidates, we used a randomized cross-validation scheme. For each cross-validation iteration, the dataset was split into 80% for model estimation and 20% for evaluation. To ensure fair comparison, we made sure that all methods use the same splits. Evaluation scores were computed for 10 cross-validation iterations and averaged. We report results for 6 evaluation measures: Pearson correlation, Kendall’s *τ*, NDCG@1, NDCG@5, NDCG@10 and Mean NDCG@10. For models which need hyper-parameter tuning, we further used 5-fold cross-validation within the train split. To obtain the best possible results, we always selected the hyper-parameters which maximize the same measure as used for evaluation. For example, for NDCG@10 results, the hyper-parameters were selected to maximize NDCG@10.

#### Parameter inference for Bayesian regression methods

Parameters of the Bayesian regression methods were estimated using variational Bayesian approaches. The algorithms for BL and EBL were proposed by [20]. The wBSR algorithm was introduced by [13]. [51] introduced a variational Bayesian algorithm for linear regression with a spike and slab prior. A difference between the algorithm for BayesC in this study and that in [51] is that the authors used importance sampling to infer the posterior distribution of *σ* ^2^, $\tau_{0}^{2}$ and *π*, whereas we inferred the factorized posteriors of *σ* ^2^ and $\tau_{0}^{2}$, and used a fixed value for *π* as described further below. The algorithms of SSVS and MIX were implemented by the second author and will be published elsewhere. Phenotypic values were standardized prior to training. All Bayesian regression methods were performed with a program written in C.

#### Model estimatation for other methods

For random forests and GBRT, we used implementations provided in the scikit-learn Python package [52, 53]. For ridge and RKHS regression, we used the R package *rrBLUP*[54]. For McRank and LambdaMART, we used the source code available at https://github.com/mblondel/ivalice. For RankSVM, we solved the objective function with *r* = 2 by L-BFGS [40]. We set the maximum number of iterations to 500.

#### Hyper-parameter tuning

For BL, we tested five values, 0.1, 1, 10, 30 and 100, for *ϕ*. For *ω*, we tested six log-spaced values from *ϕ*/20*p* to 5*ϕ*, where *p* is the number of markers. Because $\text{E}[1/\tau_{j}]=2/\lambda_{B}^{2}$ if $\tau_{j}^{2}∼InvGamma(1,\lambda_{B}^{2}/2)$, and the expectation of $\lambda_{B}^{2}$ is *ϕ*/*ω*, these values of *ϕ* and *ω* correspond to the grids of $1/\tau_{j}^{2}$ from 1/10*p* to 10, which are obtained by replacing $2/\lambda_{B}^{2}$ with *ϕ*/*ω*. In total, this corresponds to 30 possible parameter combinations. For EBL, we used the same values for *ψ* and *θ* and tested three values, 0.1, 1 and 10. For *ϕ* and *ω*, we tried the same values as for BL. Consequently, this corresponds to a total of 90 parameter combinations. For wBSR and BayesC, we fixed *ν* to 4. For *π*, we tested 5 log-spaced values from 1/*p* to 1. For *S* ^2^, we tested 10 log-spaced values from 1/20*p* to 5. Because the expectation of *InvChi*2(*ν*, *S* ^2^) is *νS* ^2^/(*ν*−2), these values of *ν* and *S* ^2^ correspond to the grids of $\sigma_{j}^{2}$ from 1/10*p* to 10. This corresponds to a total of 50 possible parameter combinations for wBSR and BayesC. For SSVS and MIX, we fixed *ν* and *π* to 4 and 0.01, respectively. For *c*, we tested 5 values from 10^−5^ to 10^−1^. The values tested for *S* ^2^ were the same as that of wBSR and BayesC. This corresponds to a total of 50 possible parameter combinations tested for SSVS and MIX.

For tree-based ensemble methods including random forests (RF), gradient boosting regression trees (GBRT), McRank and LambdaMART, we used 300 trees in the ensemble. The parameter max_features was set to 0.6, meaning that only 60% of the features are considered when searching for the best split during tree induction. This both speeds up tree induction and reduces overfitting. The maximum tree depth max_depth was chosen from {3, 5, 10}. For GBRT and LambdaMART, we chose the learning rate parameter from {0.001, 0.01, 0.1, 1.0}. For McRank, we chose the number of bins from {3, 4, …, 20}.

For ridge and RKHS regression, we used the R package *rrBLUP*[54], which can estimate the regularization and kernel parameters automatically by (restricted) maximum likelihood (i.e., without cross-validation). This approach is closely related to gaussian processes in the machine learning community [55].

For RankSVM, we set $\lambda =|P|\tilde{\lambda }$, where ∣*P*∣ is the number of preference pairs, and chose $\tilde{\lambda }$ from 15 log-spaced values between 10^−6^ and 10^6^. We use the RBF kernel *κ*(***x***_*i*_,***x***_*j*_) = exp(−*γ*‖***x***_*i*_−***x***_*j*_‖^2^). Following [54], we set $\gamma =\frac{1}{4p\sigma^{2}}$, where *p* is the number of markers. We then chose *σ* from 10 linearly-spaced values between 0 and 1.

### Cross-validation results

The general method ranking across six datasets is given in Table 1. Overall, the five best methods for each evaluation measure were as follows:

- Pearson correlation: RKHS regression, Ordinal McRank, RF, RankSVM, wBSR
- Kendall’s *τ*: RKHS regression, Ordinal McRank, RF, RankSVM, wBSR
- NDCG@1: RF, RankSVM, Ordinal McRank, RKHS regression, GBRT
- NDCG@5: Ordinal McRank, RF, RankSVM, RKHS, BL
- NDCG@10: Ordinal McRank, RKHS regression, RF, RankSVM, GBRT
- Mean NDCG@10: Ordinal McRank, RF, RKHS regression, RankSVM, GBRT

**Table 1:** General method ranking, obtained by sorting methods according to their average ranking across 6 datasets.

Method	Correlation	Kendall’s *τ*	NDCG@1	NDCG@5	NDCG@10	Mean NDCG@10
Ordinal McRank	2	2	3	1	1	1
RF	3	3	1	1	3	2
RKHS	1	1	4	4	1	3
RankSVM	4	3	2	3	4	4
GBRT	6	7	5	6	5	5
LambdaMART			8	10	8	6
Ridge	12	12	7	7	6	7
BL	7	6	5	5	8	8
MIX	11	11	10	11	10	9
SSVS	8	9	11	8	12	10
BayesC	8	8	12	9	11	11
EBL	10	10	9	12	7	12
wBSR	4	5	13	13	13	13

Detailed results for each dataset are given in Tables 2–7. For datasets comprising several traits, we report the average scores only, for the purpose of clarity. Bold numbers in parentheses indicate the ranking of the five best methods with respect to the corresponding evaluation measure. We do not include results of LambdaMART with respect to Pearson correlation and Kendall’s *τ*, since LambdaMART is a method designed to optimize NDCG.

**Table 2:** Cross-validation results on the *Arabidopsis thaliana* dataset, averaged across 3 traits.

Method	Correlation	Kendall’s *τ*	NDCG@1	NDCG@5	NDCG@10	Mean NDCG@10
RKHS	0.651 **(1)**	0.481 **(1)**	0.836	0.884 **(2)**	0.907 **(1)**	0.883 **(1)**
SSVS	0.628 **(5)**	0.470 **(2)**	0.855 **(2)**	0.885 **(1)**	0.903 **(3)**	0.881 **(2)**
MIX	0.630 **(3)**	0.468 **(3)**	0.844 **(3)**	0.872	0.899 **(4)**	0.878 **(3)**
RF	0.628 **(5)**	0.450	0.841 **(4)**	0.879 **(3)**	0.899 **(4)**	0.877 **(4)**
LambdaMART			0.814	0.870	0.904 **(2)**	0.876 **(5)**
BL	0.627	0.468 **(3)**	0.861 **(1)**	0.876	0.894	0.874
Ordinal McRank	0.636 **(2)**	0.455	0.833	0.879 **(3)**	0.899 **(4)**	0.873
BayesC	0.617	0.462	0.840 **(5)**	0.871	0.888	0.872
RankSVM	0.594	0.437	0.825	0.877	0.892	0.869
GBRT	0.619	0.442	0.802	0.863	0.896	0.866
EBL	0.625	0.468 **(3)**	0.821	0.872	0.897	0.863
wBSR	0.630 **(3)**	0.468 **(3)**	0.819	0.878 **(5)**	0.873	0.857
Ridge	0.464	0.319	0.839	0.846	0.866	0.848

**Table 3:** Cross-validation results on the Barley dataset.

Method	Correlation	Kendall’s *τ*	NDCG@1	NDCG@5	NDCG@10	Mean NDCG@10
RankSVM	0.581	0.436 **(3)**	0.816 **(1)**	0.832 **(1)**	0.850 **(1)**	0.830 **(1)**
Ordinal McRank	0.566	0.432	0.783 **(3)**	0.803 **(4)**	0.829 **(4)**	0.808 **(2)**
LambdaMART			0.729	0.824 **(2)**	0.804	0.805 **(3)**
RF	0.568	0.425	0.764 **(5)**	0.809 **(3)**	0.833 **(3)**	0.802 **(4)**
RKHS	0.604 **(1)**	0.447 **(1)**	0.766 **(4)**	0.799 **(5)**	0.834 **(2)**	0.795 **(5)**
GBRT	0.554	0.409	0.722	0.768	0.820 **(5)**	0.775
SSVS	0.585 **(4)**	0.428	0.718	0.771	0.809	0.774
Ridge	0.572	0.421	0.700	0.756	0.820 **(5)**	0.763
BL	0.581	0.432	0.790 **(2)**	0.782	0.813	0.762
MIX	0.582 **(5)**	0.434 **(5)**	0.745	0.765	0.805	0.759
BayesC	0.593 **(2)**	0.438 **(2)**	0.682	0.765	0.814	0.756
EBL	0.578	0.419	0.764 **(5)**	0.744	0.808	0.746
wBSR	0.592 **(3)**	0.435 **(4)**	0.492	0.758	0.768	0.733

**Table 4:** Cross-validation results on the Maize dataset.

Method	Correlation	Kendall’s *τ*	NDCG@1	NDCG@5	NDCG@10	Mean NDCG@10
Ordinal McRank	0.427 **(4)**	0.298 **(3)**	0.762 **(3)**	0.783 **(1)**	0.795 **(1)**	0.773 **(1)**
GBRT	0.419 **(5)**	0.283 **(4)**	0.793 **(1)**	0.721	0.768 **(4)**	0.768 **(2)**
RankSVM	0.445 **(1)**	0.317 **(1)**	0.780 **(2)**	0.771 **(3)**	0.794 **(2)**	0.765 **(3)**
RF	0.444 **(2)**	0.309 **(2)**	0.726 **(4)**	0.763 **(4)**	0.776 **(3)**	0.757 **(4)**
LambdaMART			0.696	0.697	0.740	0.741 **(5)**
Ridge	0.403	0.255	0.716 **(5)**	0.740	0.743	0.741 **(5)**
MIX	0.361	0.229	0.603	0.702	0.733	0.739
RKHS	0.431 **(3)**	0.278 **(5)**	0.675	0.736	0.761 **(5)**	0.737
BL	0.383	0.241	0.626	0.755 **(5)**	0.725	0.725
BayesC	0.393	0.242	0.582	0.773 **(2)**	0.744	0.708
wBSR	0.404	0.258	0.360	0.716	0.719	0.705
SSVS	0.398	0.240	0.592	0.734	0.730	0.687
EBL	0.390	0.236	0.654	0.725	0.739	0.644

**Table 5:** Cross-validation results on the Rice dataset, averaged across 14 traits.

Method	Correlation	Kendall’s *τ*	NDCG@1	NDCG@5	NDCG@10	Mean NDCG@10
RF	0.719 **(2)**	0.535 **(2)**	0.930 **(1)**	0.941 **(1)**	0.946 **(1)**	0.941 **(1)**
Ordinal McRank	0.717 **(3)**	0.533 **(3)**	0.921 **(2)**	0.940 **(3)**	0.946 **(1)**	0.940 **(2)**
RankSVM	0.702	0.525	0.921 **(2)**	0.937 **(4)**	0.944 **(5)**	0.937 **(3)**
GBRT	0.713 **(5)**	0.527 **(5)**	0.917 **(5)**	0.941 **(1)**	0.945 **(3)**	0.937 **(3)**
RKHS	0.720 **(1)**	0.536 **(1)**	0.916	0.937 **(4)**	0.945 **(3)**	0.936 **(5)**
Ridge	0.694	0.511	0.909	0.932	0.940	0.930
LambdaMART			0.920 **(4)**	0.931	0.934	0.929
BL	0.714 **(4)**	0.529 **(4)**	0.896	0.924	0.938	0.924
EBL	0.708	0.526	0.889	0.921	0.935	0.922
MIX	0.676	0.502	0.886	0.923	0.933	0.920
SSVS	0.686	0.506	0.889	0.918	0.932	0.916
BayesC	0.688	0.505	0.899	0.918	0.932	0.914
wBSR	0.693	0.508	0.830	0.909	0.925	0.904

**Table 6:** Cross-validation results on the Wheat (CIMMYT) dataset, averaged across 4 traits.

Method	Correlation	Kendall’s *τ*	NDCG@1	NDCG@5	NDCG@10	Mean NDCG@10
RKHS	0.503 **(1)**	0.359 **(1)**	0.698 **(2)**	0.752 **(1)**	0.780 **(1)**	0.748 **(1)**
Ordinal McRank	0.486 **(2)**	0.351 **(2)**	0.688 **(5)**	0.745 **(2)**	0.764 **(2)**	0.740 **(2)**
RF	0.482 **(3)**	0.346 **(3)**	0.697 **(3)**	0.739 **(3)**	0.760 **(3)**	0.736 **(3)**
RankSVM	0.463	0.329 **(5)**	0.713 **(1)**	0.718	0.760 **(3)**	0.733 **(4)**
BL	0.454	0.314	0.671	0.718	0.751	0.733 **(4)**
GBRT	0.472 **(4)**	0.331 **(4)**	0.690 **(4)**	0.725 **(4)**	0.757 **(5)**	0.732
Ridge	0.451	0.310	0.651	0.724 **(5)**	0.755	0.719
BayesC	0.464	0.320	0.650	0.697	0.732	0.711
SSVS	0.463	0.319	0.654	0.707	0.728	0.711
MIX	0.458	0.318	0.658	0.709	0.735	0.706
EBL	0.448	0.312	0.675	0.690	0.735	0.699
LambdaMART			0.636	0.695	0.715	0.697
wBSR	0.465 **(5)**	0.322	0.524	0.627	0.677	0.666

**Table 7:** Results on the Wheat (Pérez-Rodríguez) dataset, averaged across 2 traits.

Method	Correlation	Kendall’s *τ*	NDCG@1	NDCG@5	NDCG@10	Mean NDCG@10
RKHS	0.662 **(2)**	0.448 **(2)**	0.981 **(2)**	0.979 **(3)**	0.982 **(1)**	0.981 **(1)**
RankSVM	0.649 **(4)**	0.448 **(2)**	0.969	0.981 **(1)**	0.982 **(1)**	0.980 **(2)**
RF	0.658 **(3)**	0.438 **(4)**	0.973 **(5)**	0.980 **(2)**	0.981 **(4)**	0.979 **(3)**
Ordinal McRank	0.665 **(1)**	0.452 **(1)**	0.975 **(4)**	0.979 **(3)**	0.982 **(1)**	0.979 **(3)**
Ridge	0.602	0.398	0.982 **(1)**	0.979 **(3)**	0.980 **(5)**	0.979 **(3)**
GBRT	0.649 **(4)**	0.434 **(5)**	0.976 **(3)**	0.977	0.980 **(5)**	0.976
LambdaMART			0.973 **(5)**	0.976	0.975	0.975
BL	0.608	0.398	0.972	0.977	0.975	0.974
EBL	0.596	0.387	0.959	0.969	0.975	0.969
BayesC	0.586	0.374	0.953	0.969	0.972	0.967
MIX	0.568	0.365	0.944	0.963	0.974	0.964
SSVS	0.570	0.373	0.936	0.964	0.971	0.961
wBSR	0.578	0.381	0.915	0.951	0.965	0.959

### Comparison of RKHS regression and RankSVM

**Fig 1:**
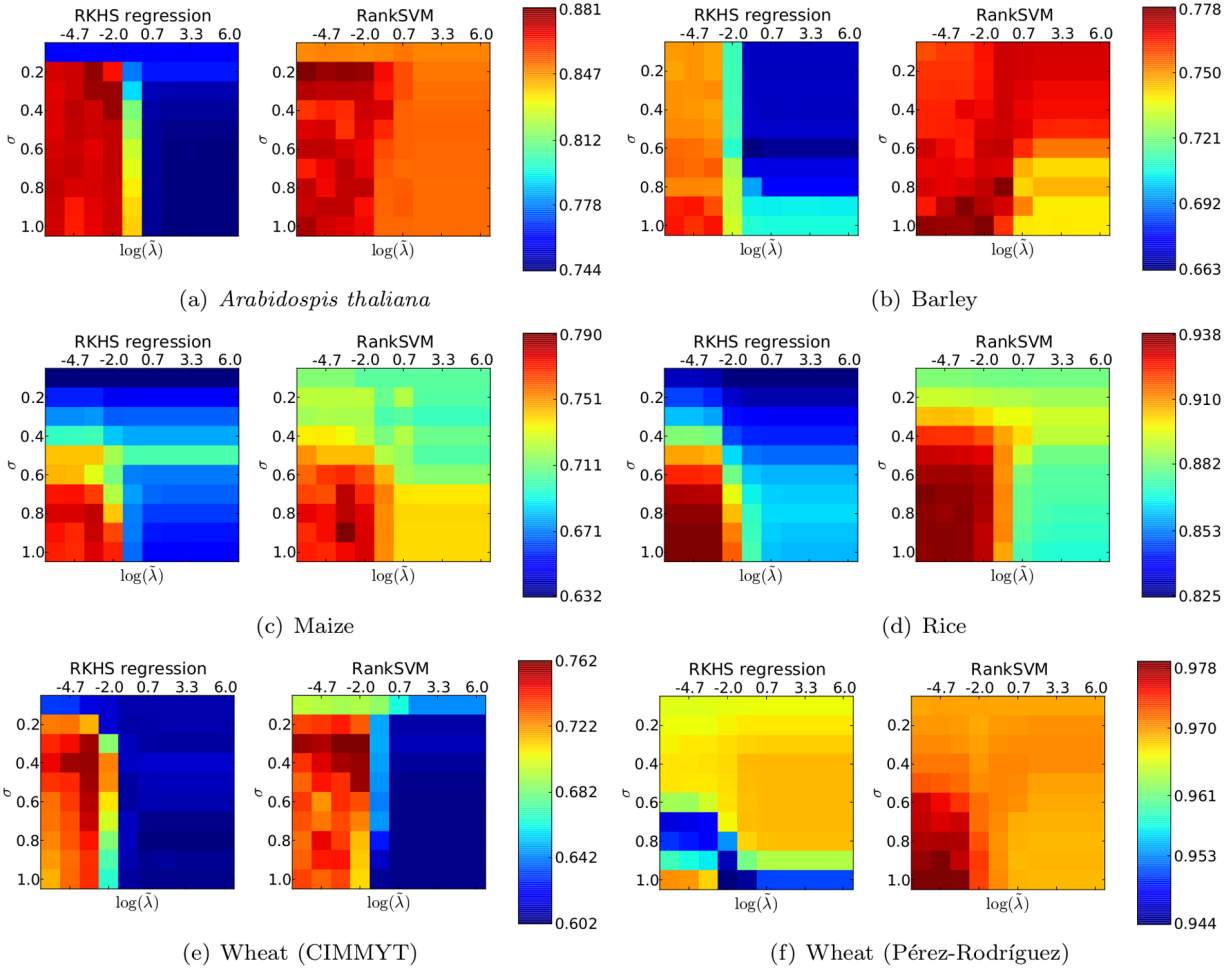
Comparison of RKHS regression and RankSVM when using the RBF kernel with parameter $\gamma =\frac{1}{4p\sigma^{2}}$ and when varying the regularization parameter $\lambda =N\tilde{\lambda }$, where *N* = *n* (RKHS regression) or *N* = ∣*P*∣ (RankSVM). The scores indicated are the test Mean NDCG@10 averaged over 10 CV iterations and across all traits.

We compared RKHS regression and RankSVM when varying the regularization and RBF kernel hyper-parameters. Heatmaps indicating Mean NDCG@10 for various hyper-parameter combinations are shown in Fig 1. Overall, our results reveal an interesting trend: RankSVM achieves better Mean NDCG@10 than RKHS regression for many different hyper-parameter settings. That is, RankSVM appears to be much more robust than RKHS regression to hyper-parameter choice.

### Experiments with tree-based ensemble methods

#### Effect of the learning rate and number of trees on GBRT

**Fig 2:**
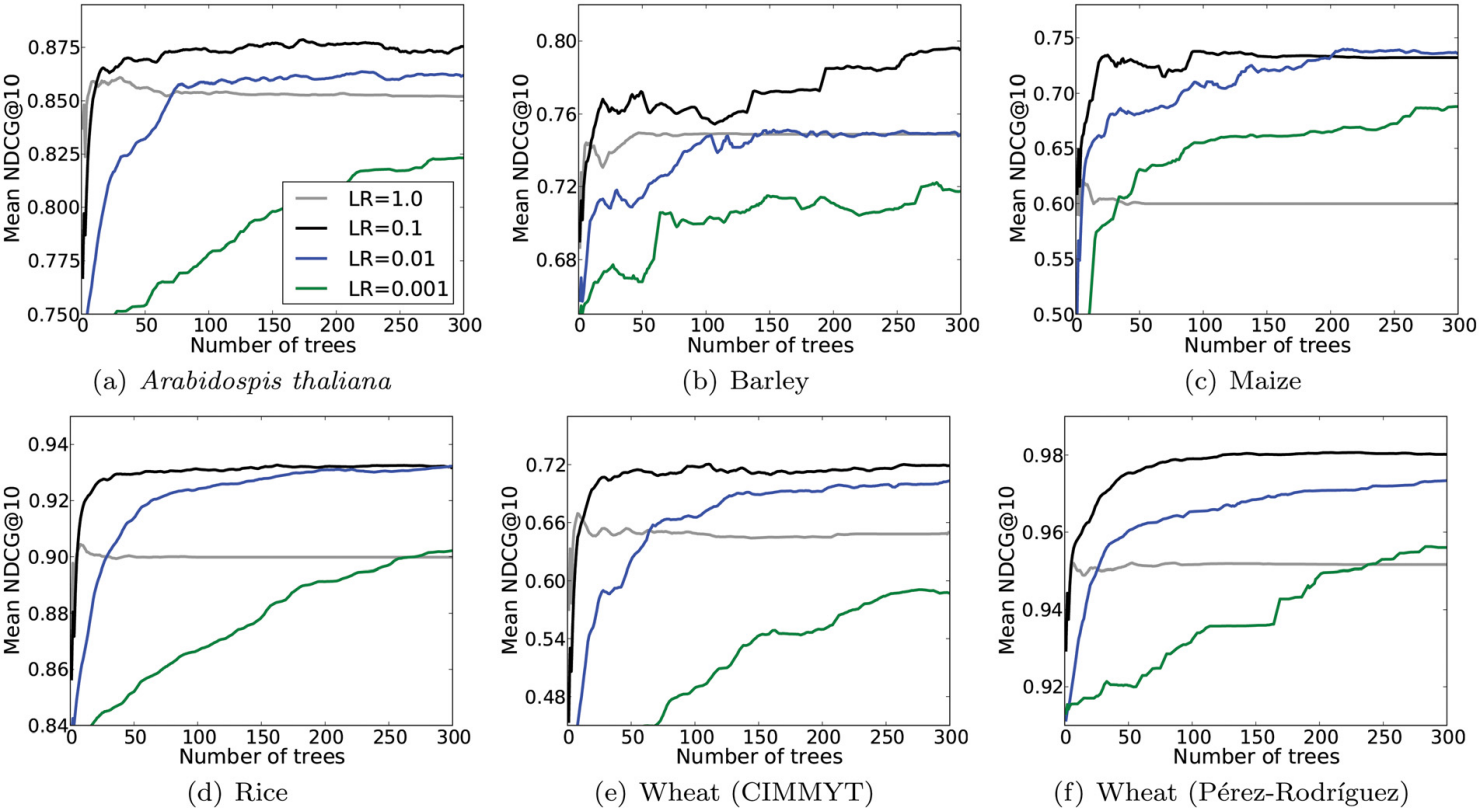
Effect of the learning rate (LR) parameter on GBRT when varying the number of trees. The scores indicated are the test Mean NDCG@10 averaged over 10 CV iterations and across all traits.

An important parameter to prevent overfitting in GBRT is the learning rate parameter. This parameter controls the importance given to the trees added at each stage of the algorithm. We compared GBRT with learning rates {1.0, 0.1, 0.01, 0.001} when varying the number of trees. Results for Mean NDCG@10 are shown in Fig 2. Our results show that, in order to obtain optimal accuracy, the learning rate must be set neither too large nor too small. This is because large values overfit the training set while small values underfit it. Except on the Maize dataset, the best learning rate was 0.1. This is value is therefore a good rule of thumb for a practical application of GBRT to GS.

#### Effect of the number of bins on McRank

**Fig 3:**
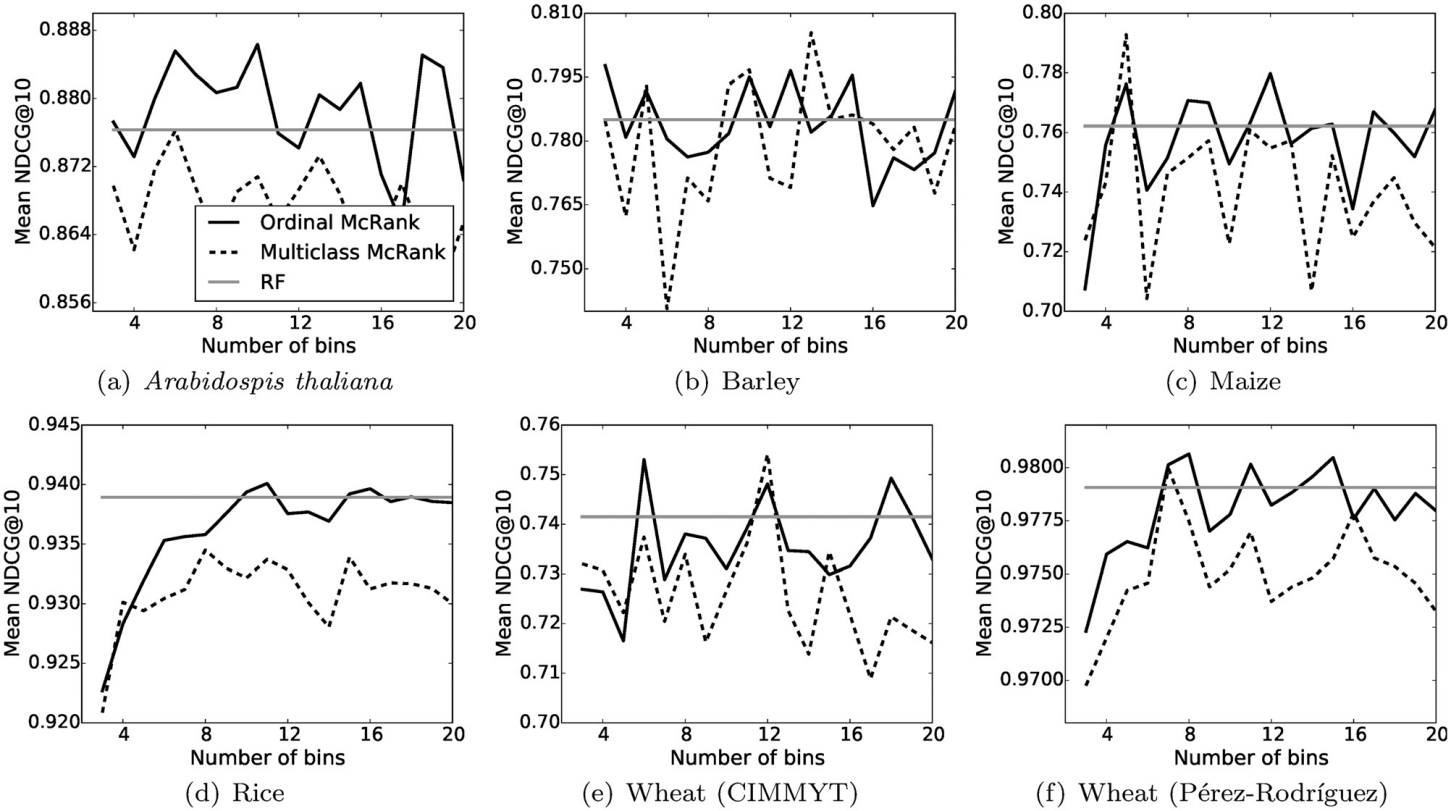
Effect of the number of bins on multiclass and ordinal McRank. The straight line indicates the results of RF for comparison. The scores indicated are the test Mean NDCG@10 averaged over 10 CV iterations and across all traits.

Since McRank assumes that 𝓨 is a finite set, we need to discretize the continuous training trait values. To do so, we divided the training trait values into *B* equal-width bins and computed their means *b*_1_, *b*_2_, …, *b*_*B*_. Then, we replaced training trait values *y*_*i*_ by the mean value of the bin they belong to. Although this discretization might sound like a loss of information, it can be beneficial when the goal is to maximize NDCG on unseen data, i.e., data that does not belong to the training set. We compared the Mean NDCG@10 of multiclass and ordinal McRank when varying the number of bins. The number of trees used was fixed to 300. Results are shown in Fig 3. For comparison, we also indicate the results of RF regression. Our results clearly show the superiority of ordinal McRank over multiclass McRank. Therefore, modeling the cumulative probabilities, as done in ordinal McRank, seems clearly beneficial.

### Comparison of evaluation measures

Table 1 shows that the best methods tend to vary depending on the evaluation measure used. To quantify the similarity (or lack thereof) between evaluation measures, we computed their Spearman’s correlation coefficient, also known as Spearman’s *ρ*, which is a measure of how well their rankings agree. The results, given in Table 8, indicate several interesting trends.

**Table 8:** Spearman’s rank correlation coefficient of evaluation measures averaged across 6 datasets.

Method	Correlation	Kendall’s *τ*	NDCG@1	NDCG@5	NDCG@10	Mean NDCG@10
Correlation	-	0.899	0.604	0.774	0.775	0.744
Kendall’s *τ*	0.899	-	0.564	0.665	0.674	0.664
NDCG@1	0.604	0.564	-	0.811	0.827	0.901
NDCG@5	0.774	0.665	0.811	-	0.923	0.920
NDCG@10	0.775	0.674	0.827	0.923	-	0.962
Mean NDCG@10	0.744	0.664	0.901	0.920	0.962	-

First, Pearson correlation and Kendall’s *τ* correlate poorly with NDCG@k when *k* is small. This is not surprising since Pearson correlation and Kendall’s *τ* are evaluation measures for global ranking which treat all individuals equally, regardless of their importance. This confirms that one should not use these measures for evaluation and hyper-parameter selection when one is mostly concerned with ranking accuracy at the top.

Second, and more surprisingly, Pearson correlation was more correlated with NDCG@k than Kendall’s *τ*. This suggests that a method designed to maximize Pearson correlation might perform better than a pairwise ranking method.

Finally, the most correlated measure with NDCG@1, NDCG@5 and NDCG@10 was Mean NDCG@10. This suggests that choosing a model which maximizes Mean NDCG@k is a good compromise, if we want a model which works reasonably well (without retraining) at various positions *k*.

## Discussion

The choices of the position *k*, gain function *g*(*y*) and discount function *d*(*i*) used in the DCG and NDCG definitions naturally depend on the usecase. For long-lived perennial plants, such as fruit and forest trees, it is important to select good candidates at their juvenile stage (or even at their small seedling stage) for further field testing. At the same time, it is also important to select a small number *k* of candidates because selected candidates usually become parents for the next generation. If too many candidates are selected, selection intensity becomes low and it is not possible to obtain good improvement of the target trait in the next generation. For these reasons, and since field testing is typically expensive, we chose to evaluate models for small values of *k*: *k* ∈ {1, 5, 10}. In the IR literature, the exponential gain function *g*(*y*) = 2^*y*^−1 is frequently used. This is because it is often assumed that more relevant documents are exponentially more useful than irrelevant documents. However, in IR, *y* is usually a small number (say, between 1 and 5) which assesses the relevance of a document to some query. In contrast, in GS, *y* can take on much larger values depending on the trait. For this reason, we choose the linear gain function *g*(*y*) = *y*. For the discount function, a possible choice is to not use any discount at all, i.e., *d*(*i*) = 1. This amounts to completely ignore order in the top-*k* candidates. This choice is only reasonable if we are sure to conduct field testing for all *k* selected candidates. Another possible choice is $d(i)=\frac{1}{{\text{log}}_{2}(i+1)}$, which is also the most common choice in the IR literature. This discount function assigns a monotonically decreasing weight to candidates as a function of their rank. It thus takes into account the fact that a candidate is more likely to be examined if it is placed higher in the ranking. This choice is more reasonable if we need to prioritize field testing of candidates with high breeding value, for example due to budget or time constraints. For this reason, we chose $d(i)=\frac{1}{{\text{log}}_{2}(i+1)}$ in our experiments.

Trees are a non-parametric method which can approximate complex functions. However, they tend to severely overfit the training data when used alone. For this reason, trees are usually used as part of an ensemble method. Overall, we found that tree-based ensemble methods perform very well for ranking. This confirms a trend which was also observed during the Yahoo! learning to rank challenge [27]. With respect to Mean NDCG@10, Ordinal McRank, RF and GBRT achieved overall 1st, 2nd and 5th places. Tree-based ensemble methods have a number of other advantages including their ability to handle categorical variables, handle missing values without prior imputation and estimate variable importances and interactions [25, 26]. Therefore, while the GS community has until now mainly focused on ridge regression and Bayesian regression methods, we believe that tree-based ensemble methods should be considered a top contender in the context of GS.

RKHS regression achieved 1st place with respect to Pearson correlation on four out of six datasets. Therefore, our results confirm the good results of RKHS regression previously reported in the literature [10]. With respect to Mean NDCG@10, RKHS regression proved to be a very good method, achieving an overall 3rd place. However, this shows that the best method with respect to Pearson correlation is not necessarily the same as the best method with respect to NDCG or Mean NDCG.

RankSVM achieved an overall 4th place with respect to Mean NDCG@10. However, with respect to NDCG@1 and NDCG@5, RankSVM outperformed RKHS regression. On the Barley dataset, RankSVM outperformed other methods on all NDCG measures by a very large margin. Our results also show that RankSVM is less sensitive than RKHS regression to hyper-parameter choice.

LambdaMART achieved a disappointing overall 6th place with respect to Mean NDCG@10. This is worse than regular GBRT, of which LambdaMART is an extension. This is surprising, since LambdaMART achieved the leading results in the Yahoo! learning to rank challenge [27]. However, in the Yahoo! learning to rank challenge, the number of samples *n* was much greater than the number of features *p*, i.e., *n* ≫ *p*. This contrasts with GS, where typically *n* ≪ *p*. We thus hypothesize that LambdaMART does not work well in the *n* ≪ *p* setting.

Traditional (Bayesian) regression methods overall did not perform well with respect to NDCG and Mean NDCG. For example, although they were suggested as some of the best methods in the recent study of [15], BL and wBSR only achieved overall 7th and 13rd places, with respect to Mean NDCG@10. One exception where traditional regression methods performed well is the *Arabidopsis thaliana* dataset, with RKHS regression, SSVS and MIX achieving 1st, 2nd and 3rd places, respectively, with respect to Mean NDCG@10. In the *Arabidopsis* dataset, genotypes are all recombinant inbred lines (RILs) derived from two homozygous parents. Therefore, quantitative trait loci (QTL) harbored by lines (i.e., RILs) are completely bi-allelic. In contrast, in other datasets, QTL harbored by lines may have allelic variation. In fact, important agronomic traits have multiple alleles in candidate genes [56]. In this case, allelic effects cannot be represented by a single bi-allelic marker. Therefore, there may exist complex relationships between causal polymorphisms and SNPs linked to the polymorphisms. In the *Arabidopsis* dataset, the extent of linkage disequilibrium (LD) between a marker and QTL is simply related to the recombination rate between the marker and QTL. In contrast, in other datasets, the extent of LD between a marker and QTL may be affected by various other factors such as demographic history [57]. In this case, the relationship between markers and QTL becomes complex and may be difficult to model via linear regression models.

On the rice dataset, the difference between the best (RF) and worst (wBSR) methods with respect to NDCG@10 appears quite small (0.925 vs. 0.946), while it appears larger with respect to NDCG@1 (0.930 vs. 0.830). Similar trends are shown in other datasets, such as barley and wheat. We observed that the NDCG@k score typically increases as a function of *k* (although not monotonically). That is, if *k* < *r* then NDCG@k < NDCG@r will usually hold. Since NDCG@k is always between 0 and 1, this means that NDCG@k gets closer to 1 as *k* increases. This also means that the range of possible values taken by NDCG@k will typically decrease as a function of *k*. This however does not necessarily mean that small values of *k* are better for discriminating between methods. In general, *k* should be set to the number of candidates one wants to select when applying the model.

Sometimes, both ranking and regression accuracies are important. This is for example the case when predicted trait values are used to determine a selling price (e.g., determine crop price in terms of the predicted grain yield). Because of their overall good ranking accuracy, RF and RKHS regression seem like a good choice in this case. Unfortunately, RankSVM and LambdaMART cannot be used in this case, since the loss function they minimize only guarantees ranking. On the other hand, McRank can be used, since it can compute the expected trait values.

For simplicity, we assumed throughout this paper that our goal is to select candidates with a high trait value. Of course, it is easy to adapt our framework if the goal is to select candidates with low trait value instead. However, sometimes it may be necessary to select candidates with an appropriate value, rather than highest or lowest value. For example, a certain level of acidity is necessary for fruits, but excessive or insufficient acidity is not preferred. In this case, model evaluation based on mean squared error (MSE) may be more suitable.

Pearson correlation is a commonly used measure in GS because it enables to predict the response to selection (provided there is no non-genetic cause of resemblance between offspring and parents) [58]. Because the response to selection corresponds to the change of population mean, Pearson correlation is important for breeding schemes that focus on whole population improvement. This is typical in animal breeding such as dairy cattle, where a single head of cattle contributes only little to the total production gain. However, particularly in breeding of plants that can be clonally reproduced (e.g., inbred crops or graftable fruit trees), the aim is often to produce a few excellent lines, rather than improving an entire population. In this case, Pearson correlation may not always be a good choice. Our results using 4 plant species show indeed that Pearson correlation often correlates poorly with NDCG. On the other hand, we found that Mean NDCG was the most correlated with NDCG at various positions.

Our study suggests two important messages. First, ranking methods are a promising research direction in GS. Second, NDCG and Mean NDCG can be useful evaluation measures for GS, especially in plant breeding.
